# Mobile telephone provider to provider helpline: A pilot study in Cameroon

**DOI:** 10.1016/j.xagr.2025.100581

**Published:** 2025-10-24

**Authors:** Comfort Enah, Victoria Jauk, Mary Glory Ngong, Christyenne Lily Bond, Lionel Neba Ambe, Rahel Mbah Kyeng, Roland Mbole, Waldemar A. Carlo, Gregory Halle-Ekane, Pius Muffih Tih, Jeff Szychowski, Henna Budhwani, Alan Thevenet N. Tita

**Affiliations:** 1 Solomont School of Nursing, College of Health Sciences, University of Massachusetts Lowell, MA (Enah); 2 Department of Obstetrics and Gynecology, University of Alabama at Birmingham Heersink School of Medicine, Birmingham, AL (Jauk, Tita); 3 Cameroon Baptist Convention Heath Services (CBCHS), Bamenda, Northwest Region, Cameroon (Ngong, Kyeng, Mbole, Tih); 4 Institute on Digital Health and Innovation, College of Nursing, Florida State University, Tallahassee, FL (Bond, Budhwani); 5 Regional Delegation of Public Health, Northwest Region, Cameroon (Ambe); 6 Division of Neonatology, University of Alabama at Birmingham Heersink School of Medicine, Birmingham, AL (Carlo); 7 University of Buea, Molyko, Buea, Cameroon (Halle-Ekane); 8 School of Public Health, University of Alabama at Birmingham, Alabama, (Szychowski); 9 Center for Women’s Reproductive Health, University of Alabama at Birmingham Heersink School of Medicine, Birmingham, Alabama (Budhwani, Tita)

**Keywords:** intervention, maternal health, mHealth, mobile applications, neonatal health, pregnancy provider, support hotline

## Abstract

**Background:**

Despite the availability of effective strategies, countries in Sub-Saharan Africa continue to be disproportionately affected by maternal and perinatal morbidity and mortality. Mobile health interventions are recognized for their potential usefulness in addressing gaps to quality maternal and neonatal care in low-income countries, including those for health care providers.

**Objectives:**

To measure feasibility and acceptability of an adapted 24/7 mobile phone-based medical information service via telephone (mMIST) provider-to-provider intervention in one district of Cameroon.

**Study Design:**

We implemented a multimethods pilot study of mMIST in Ndop Health District in the Northwest region of Cameroon from March 2022 to February 2023. We projected on average one call every 2 days as an acceptable demand level, and at least 70% of providers rated the mMIST intervention as satisfactory for acceptability. We evaluated the feasibility of collecting administrative data on maternal and neonatal morbidity and mortality for the assessment of mMIST on clinical outcomes.

**Results:**

Thirty front line and on-call expert maternal healthcare workers who staffed the mMIST answering line and 76 peripheral maternity providers in the Ndop Health District were trained to use the system. A total of 3991 births were reported during the mMIST pilot year. Monthly calls ranged from 2 in the first month to a high of 28 calls by the 6th month, with an average call volume of 14 per month. Forty-six out of 48 providers (96%; 95% confidence interval [78,95]) were satisfied with mMIST. Providers reported that intermittent electricity and internet connectivity issues were the main barriers to usage. We found that it was feasible to obtain administrative data on relevant neonatal and maternal outcomes in the district but inconsistencies in local reporting prevented reliable comparisons.

**Conclusion:**

Overall, the mMIST intervention was feasible to implement and was acceptable to maternal healthcare providers who staffed or used the intervention. Adaptation to local context, engagement of, and buy-in from multiple stakeholders in our formative work contributed to feasibility and promising findings to inform larger scale evaluation of the intervention.

**AJOG Global Reports at a Glance**

**Why was the study conducted?**

To pilot a newly adapted 24/7 mobile phone-based medical information service via telephone (mMIST) provider-to-provider support intervention among maternity health workers in one district of Cameroon to determine feasibility and acceptability

**Key findings**

The mMIST intervention was feasible to implement and was acceptable to maternal healthcare providers who staffed or used the intervention

Adaptation to local context, engagement of, and buy-in from multiple stakeholders in our formative work contributed to feasibility and promising findings

**What does this study add to what is known?**

The study adds support to the potential usefulness of mobile health interventions in low-income countries. The mMIST is a promising solution for healthcare improvement that is geographically scalable and if successfully evaluated could potentially be applicable to other disciplines

## Introduction

The 2030 Sustainable Development Goals prioritize the improvement of maternal health outcomes.^1^ In addition, for multiple decades, the World Health Organization (WHO), and other global health care organizations have drawn attention to reducing maternal and neonatal mortality rates as important public health focal areas.^1^^,^^2^ Although progress has been made in improving maternal health outcomes with the maternal mortality rate dropping 38% globally between 2000 and 2017,^3^ these gains were not uniform across countries. For example, a woman’s lifetime risk of maternal death is more than a thousand times higher for women in low-income countries compared to women in high-income countries.^3^ In addition, 94% of all maternal death still occur in low-income countries.^3^ Lack of timely access and poor quality of prenatal care in low- and middle-income countries (LMIC) have been identified as major contributors.^1^^,^^4^ Cameroon remains one of the counties that are disproportionately affected with maternal mortality as high as 438 per 100,000 in 2020 and neonatal mortality rates as high as 26.2 per 1000.^5^

Mobile health (mHealth) evidence-based interventions are increasingly recognized for their potential usefulness in addressing maternal and neonatal health disparities.^6^, ^7^, ^8^, ^9^ In particular, provider focused mHealth interventions for obstetric care have shown promise in several sub- Saharan countries including Rwanda^8^ and Ethiopia.^10^ Using mHealth approaches can circumvent barriers to access and provide greater reach into communities that are often characterized by poor transportation infrastructure and geographically dispersed populations.^8^, ^9^, ^10^, ^11^, ^12^, ^13^, ^14^, ^15^, ^16^ mHealth approaches gained increased prominence during the height of the COVID- 19 pandemic.^13^, ^14^

The availability of cell phones in LMICs, including Cameroon, presents an opportunity to deliver mHealth interventions to remote areas with limited access to land lines and knowledge of evidence-based interventions.^11^^,^^17^, ^18^ In Cameroon, cell phone coverage is estimated to be at 86.3%^19^ and based on our formative work, all maternal healthcare facilities and healthcare providers have access to cell phones. However, research on mHealth interventions in Cameroon remains very limited, and have focused on text messages for HIV prevention, sexual health, and TB prevention.^20^ Even though mHealth interventions in obstetric care have been evaluated in Sub Saharan Africa,^8^^,^^11^ few have focused on 24/7 mobile phone-based provider-to-provider information support hotlines and none have been evaluated in Cameroon.

In Alabama, a medical information service via Telephone (MIST), established in the 1960s (using land lines), continues to be a highly demanded service to provide real-time subspecialty support to community physicians providing care at the bedside. This intervention was recognized as a Time Magazine Innovation of the Year in 1969. The MIST system was chosen for adaptation because at the time of planning for this project, even though some intervention showed promise in LMIC, few had demonstrated high demand and effectiveness. In addition, U.S based members of our team had extensive experience with the MIST system which continues to enjoy a high level of demand from rural providers to date. We sought to adapt MIST into a mobile phone-based solution (mMIST) for provider-to-provider support in low-income countries. This feasibility and acceptability assessment of the mMIST intervention was part of a multiphased study conducted with multiple stakeholders that began with formative qualitative work to match the context in Cameroon.^17^^,^^18^ Findings from qualitative interviews of healthcare workers, previously pregnant women, and mobile phone service providers were used to adapt the University of Alabama at Birmingham (UAB) MIST system to the new contextually relevant mMIST, set up the mMIST system in a central location, test the system’s functionality and add accommodations to ensure a continuous power supply and 24/7 human resource coverage.^17^ In our formative work, we also established long term partnerships with local governmental, nongovernmental, and cellphone companies that were leveraged in the pilot phase.^17^ Detailed protocols for the formative phase of the parent study and the structural barriers faced by pregnant women in Cameroon are reported elsewhere.^17^^,^^18^ Herein, we implemented a feasibility and acceptability study of the adapted mMIST in one district in Cameroon.

## Materials and methods

We implemented a multimethods pilot study of the newly adapted mMIST ) in Ndop Health District in the Northwest region of Cameroon, applying the ADAPT-ITTmodel^21^ (a systematic method for adapting evidence-based interventions in novel context) from March 2022 to February 2023.The Ndop Health District was chosen based on the recommendation of the local regional Ministry of Health authorities, due to its central location within the region. The district has an approximate 4000 to 5000 live births each year. All facilities in the district had at least one qualified maternity care provider. The primary focus of the pilot study was on demand and acceptability, ^22^ and we assessed utilization and satisfaction. Components of feasibility were assessed using multiple sources of data including mMIST logs of numbers and content of calls and interviews with peripheral providers. We also evaluated the feasibility of collecting administrative data. All maternity care providers from the healthcare facilities in the district were targeted to participate.

### mMIST deployment and training

**Figure 1:**
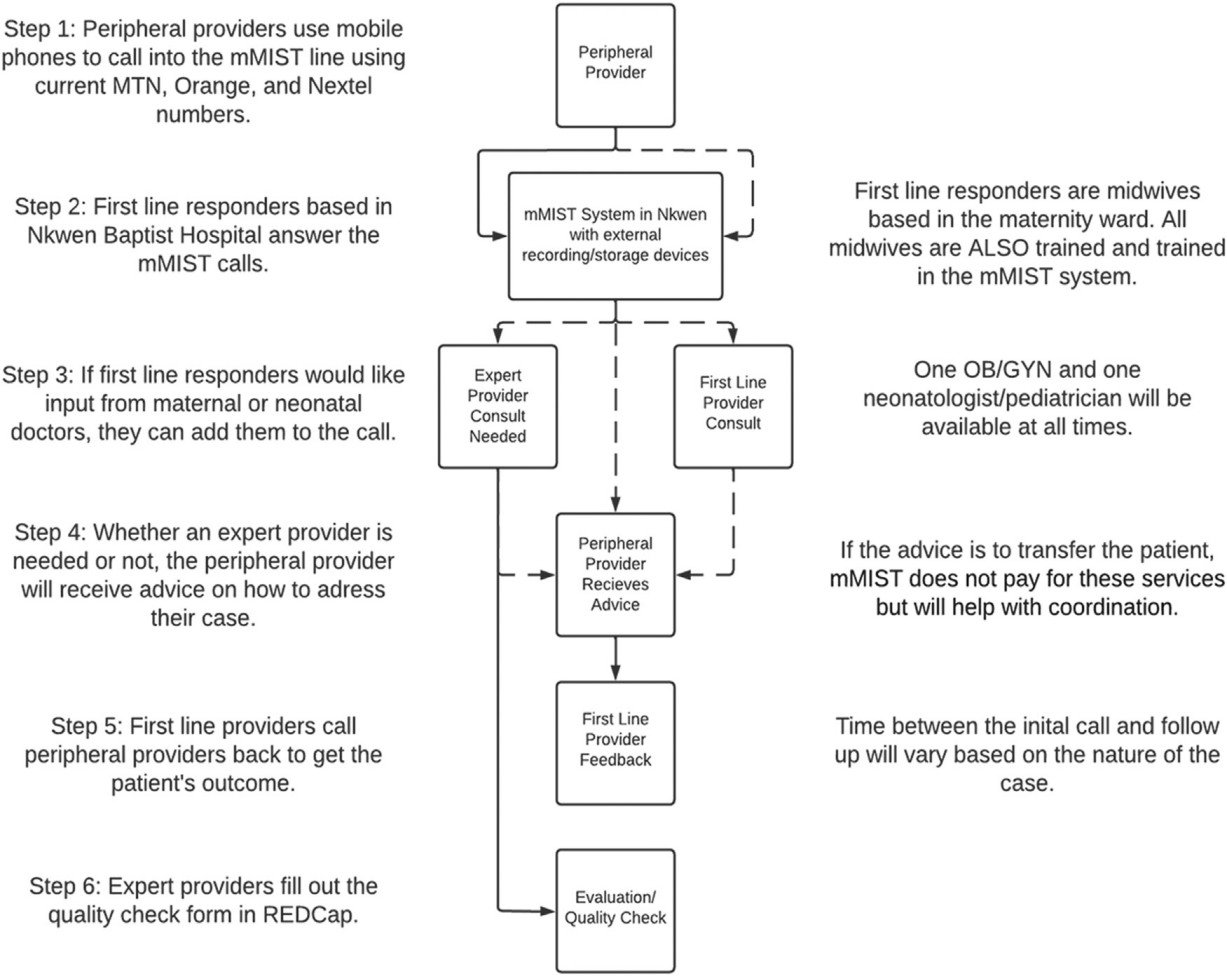
mMIST structure and call flow.

mMIST technology was deployed at the Nkwen Cameroon Baptist Convention Health System (CBCHS) Hospital in the regional capital city of Bamenda and mobile handphones stationed in the labor and delivery unit. Figure 1 depicts the structure of the mMIST system. Tailored training was provided to mMIST health care workers including expert obstetrics and gynecological providers (OB/GYNS) and pediatricians, first line responders (trained nurse midwives), who answered the mMIST line 24/7, and peripheral providers who were maternity workers who called into the system for expert advice. Peripheral providers primarily consisted of nurses and midwives. In a very few rural areas, peripheral providers also include physicians with no maternal or neonatal training and skilled birth attendants. First line providers were nurse midwives purposively recruited from the Nkwen Cameroon Baptist Convention Health Services (CBCHS) Hospital based on their training in Advanced Life Support in Obstetrics (ALSO). First line providers were provided with a set of checklists based on the WHO, ALSO and local guidelines for reference when responding to calls. Expert providers were physicians with specialist or subspecialist training except 2 who were nurses with additional specialist training in neonatal care. In addition, all expert providers were experienced obstetric and neonatal providers purposively selected from multiple governmental and nongovernmental tertiary health facilities across the country. Applying a train-the-trainer model, lead peripheral providers were provided central training by the mMIST team and then given training materials and relevant fliers to train their maternity providers (eg, physicians, nurses, midwives, skilled birth attendants) at peripheral units. Information on how to access and use mMIST was posted at each unit.

### Outcomes

Primary outcomes were mMIST utilization (demand) quantified as number of calls during the pilot and acceptability quantified as percent of mMIST providers reporting satisfaction. Demand was assessed using an inventory system of the number of times peripheral providers access mMIST. The duration of calls was documented in categories of less than 5 minutes, 5 to 10 minutes, and more than 10 minutes. Acceptability was assessed through satisfaction surveys (see Appendix A) that were deployed online using a Qualtrics link and follow-up interviews. The survey link was shared with all 16 first line and 76 peripheral providers. The survey consisted of 8 closed-ended questions with the options (agree, disagree, and neither agree nor disagree) and one open-ended question (ways to improve on the mMIST). The survey was scored by the percentage of respondents who agreed with satisfaction survey statements. Feedback from first-line providers on the quality and relevance of the training was also assessed using a satisfaction survey form. Content analyses were used to summarize responses on suggestions on ways to improve on mMIST. A team consisting of investigators, expert providers and first line responders audited a random sample of taped calls to evaluate accuracy of reports.

Administrative data collection of maternal and perinatal deaths, and morbidity occurred on a rolling basis. Data collection followed routine processes used throughout the Northwest Region to report reproductive health data. Data from each health facility in the district was reported to the regional health authorities monthly, and these data were then entered into a database maintained by the regional Ministry of Health. The regional authorities of the Ministry of Health provided a copy of the database to the study team for analysis. We calculated a composite outcome comprised of maternal deaths, stillbirths registered, and neonatal deaths (0 to 28 days). We also examined major causes of death such as postpartum hemorrhage (requiring blood transfusion or hysterectomy), eclampsia/severe preeclampsia, sepsis/infections, neglected obstructed labor >12 hours after complete cervical dilation or secondary arrest, and preterm birth.

### Sample size

We focused on one district to gauge feasibility and acceptability to further adapt mMIST and related systems prior to large-scale implementation in the region. All maternity workers in the designated Ndop pilot health district were targeted for training on study protocols. Considering ∼5000 deliveries per year, we estimated 96 to 115 pregnancies weekly in the planning phase. We projected on average a minimum of 1 call every 2 days to the mMIST system as an acceptable demand level based on our experience with managing obstetrics emergencies using the original MIST system. We expected that from the survey, at least 70% of providers will find mMIST acceptable based on other trials in Sub-Saharan Africa.^23^ Therefore, the projected number of calls and estimated 50 or more providers involved in mMIST during the pilot allowed us to explore feasibility and acceptability outcomes.

### Data management and analysis

A database was developed to log call information. Data collected through a log (study form) included date, time, call duration, caller type (eg, nurse, midwife, physician, etc.), location, patient type (eg, pregnant, postpartum, or baby), call reason, respondent (eg, maternity worker, specialist expert, both), recommendation, and follow-up on advice. Provider demand was quantified by the total number of calls from all peripheral providers into the mMIST system. Satisfaction survey and follow up interview data was descriptively analyzed to gauge acceptability and feasibility.

The administrative outcome data used in the pilot were aggregated at the site level and overall, for the entire pilot district. The analysis of outcomes focused on health district data from all participating maternity health units. In additional analysis, we restricted the data to one specific hospital, the referral hospital in the region, which alone was responsible for nearly 50% of the mMIST calls received. Statistical significance was set at *P* < .05. Sensitivity analyses assessed the robustness of regional findings using data from the primary intervention hospital. All analyses were conducted using SAS version 9.4.

This study was evaluated and approved by the CBCHS Institutional Review Board (IRB2020-49) and the University of Alabama at Birmingham (UAB) Institutional Review Board (IRB-300006254).

## Results

The mMIST pilot was implemented in the Ndop Health District, Northwest region of Cameroon, during a one-year period between March 2022 and February 2023. A total of 30 health care workers (14 expert obstetricians and pediatricians and 16 first line responders) were trained to operate the mMIST system. A total of 76 peripheral providers at maternity health facilities in the Ndop Health District were trained using the train-the-trainer model. The monthly number of mMIST calls increased over time from 2 calls in the first month to 28 calls in the 6th month with an average monthly call volume of 14. While there were variations in demand over time, we met feasibility goals of averaging a minimum of 1 call every 2 days as an acceptable demand level in the district. Calls were received from 17 unique health facilities, with half of the calls originating from the single referral hospital in the district. Table 1 shows the number of facilities that used the system and number of calls received each month. The majority (53%) of calls lasts 10 to 15minutes, with 28% of calls lasting less than 5 minutes and 19% of calls lasting more than 15minutes. This duration was reasonable based on the streamlined communication training provided to study participants, and our experience with managing obstetric emergencies using the MIST system. The first line provider addressed most calls; only 30% of the calls were referred to expert provider consultation. Most calls were focused on maternal health issues (80%) compared to neonatal issues (20%). The top reasons for maternal calls included abnormal labor (28%), infections (23%), hypertensive disorders (12%), miscarriage (9%), fetal malpresentation (8%), and PROM (8%). Neonatal calls primarily focused on asphyxia (35%), infection (30%), prematurity (26%), and seizures (9%). The random samples of recorded calls that were audited were found to contain timely, accurate, and contextually relevant information that was useful to the peripheral providers who called into the system.

**Table 1:** Number of health facility calls to the mMIST per month

	Number of calls	Number of facilities
March	2	2
April	8	7
May	8	5
June	20	4
July	12	4
August	28	6
September	14	4
October	16	6
November	9	6
December	20	7
January	10	4
February	10	4

Our satisfaction survey had a response rate of 52% and findings from a total of 48 providers (33 peripheral and 15 first line providers) indicated that 96% (95% confidence interval [78,95]) of providers (46/48) were satisfied with the mMIST (exceeding the projected 70%). All respondents reported that they understood the purpose of mMIST, and 98% agreed mMIST was helpful in the provision of care. Table 2 depicts additional details on respondents’ agreement with satisfaction statements including 95% confidence intervals. The remaining responses were neutral (neither agree nor disagree). In addition, all expert providers and first line providers were retained during the duration of the pilot study. Content analyses of open-ended questions indicated that 82% of peripheral providers reported that local intermittent internet connectivity and electrical power issues were the main barrier to usage. Ten percent of respondents stated that the system worked well and had no recommended changes, 4% wanted the system to be open to providers outside of the study district and 4% wanted regular training to reinforce understanding of the system. Follow-up phone interviews of peripheral providers in facilities with no calls to the mMIST system indicated that these facilities had not encountered complicated deliveries that required additional guidance during the pilot period. First line providers reported that the mMIST system was easily incorporated into their workflow, offered opportunities to learn from specialists and increased their confidence in the care they provided to pregnant women and neonates.

**Table 2:** mMIST provider survey responses (N = 48)

Variable	Agree	Wilson 95% CI
Understand the purpose of mMIST, n (%)	48 (100%)	93% - 100%
mMIST is easy to use, n (%)	36 (75%)	61% - 85%
mMIST is helpful in providing care to patients, n (%)	47 (98%)	89% - 100%
mMIST training was good, n (%)	46 (96%)	86% - 99%
Satisfied with mMIST, n (%)	46 (96%)	78% - 95%

*CI*, confidence interval.

A total of 4677 women were delivered in the Ndop district in the year preceding the introduction of mMIST (March 2021-Februrary 2022); 3991 births were reported during the mMIST pilot year. The frequency of selected primary and secondary adverse pregnancy outcome components are shown in Table 3. Despite more labor complications in the mMIST pilot period, the frequencies of the composite outcome (composite maternal and perinatal death) did not differ significantly between period (1.2% vs 0.1%). The respective frequencies of maternal deaths (0.2% vs 0.03%) was lower during the mMIST pilot year, but stillbirths (0.7% vs 0.7%) and neonatal deaths (0.4% vs 0.2%) did not differ between periods. Among secondary outcomes some appeared higher during the mMIST pilot than prior: prolonged/obstructed labor (2.5% vs 3.8%), uterine rupture cases (0.09% vs 0.6%), abortion complications (1.2% vs 2%) and premature births (2% vs 2.6%). When we restricted the analysis to just the Ndop District Hospital, the only referral hospital in the pilot district, which accounted for about 50% of calls, similar patterns to the overall were observed (Table 4). More depth occurred in the district hospital, which may indicate that high risk cases were appropriately being referred to the district hospital, thereby increasing mortality there during the pilot period. In additional assessments of the data from the pilot, and the year preceding the pilot we observed that there were marked variations in the reporting of several outcomes and related denominators that likely reflected sub-optimal data collection practices and inaccuracies.

**Table 3:** Outcomes pre- and during the intervention period

Variable	Total	Pre Pilot	During Pilot	*P*-value
Number of women delivered, n	8668	4677	3991	
Composite outcome, n (%)^a^	95 (1.1%)	57 (1.2%)	38 (1%)	.235
Maternal deaths, n (%)	9 (0.1%)	8 (0.2%)	1 (0.03%)	.044
Stillbirths registered, n (%)	61 (0.7%)	32 (0.7%)	29 (0.7%)	.814
Neonatal deaths (0 to 28 days), n (%)	25 (0.3%)	17 (0.4%)	8 (0.2%)	.158
Labor complications				
Prolonged labor/obstructed labor, n (%)	271 (3.1%)	118 (2.5%)	153 (3.8%)	<.001
Uterine ruptures, n (%)	26 (0.3%)	4 (0.09%)	22 (0.6%)	<.001
Abortion complications, n (%)	134 (1.5%)	55 (1.2%)	79 (2%)	.003
Premature live births, n (%)	196 (2.3%)	92 (2%)	104 (2.6%)	.046
Other complications				
Hemorrhage, n (%)	332 (3.8%)	168 (3.6%)	164 (4.1%)	.211
Pre eclampsia/eclampsia, n (%)	56 (0.6%)	29 (0.6%)	27 (0.7%)	.744
Puerperal infections, n (%)	160 (1.8%)	95 (2%)	65 (1.6%)	.165
Obstetric complications treated or referred, n (%)	464 (5.4%)	230 (4.9%)	234 (5.9%)	.051
C-sections performed, n (%)	695 (8%)	383 (8.2%)	312 (7.8%)	.526
Death following abortion, n (%)	7 (0.08%)	5 (0.1%)	2 (0.05%)	.463
Live births < 2500 g, n (%)	193 (2.2%)	112 (2.4%)	81 (2%)	.251

a Composite comprised of maternal deaths, stillbirths registered, and neonatal deaths (0 to 28 days). Prepilot, January 2021 to February 2022; During pilot, March 2022 to February 2023.

**Table 4:** Outcomes pre- and during the intervention period for NDOP district hospital

Variable	Total	Pre Pilot	During Pilot	*P*-value
Number of women delivered, n	725	401	324	
Composite outcome, n (%)*	32 (4.4%)	10 (2.5%)	22 (6.8%)	.005
Maternal deaths, n (%)	0 (0%)	0 (0%)	0 (0%)	-
Stillbirths registered, n (%)	29 (4.0%)	10 (2.5%)	19 (5.9%)	.021
Neonatal deaths (0 to 28 days), n (%)	3 (0.4%)	0 (0%)	3 (0.9%)	.089
Labor complications				
Prolonged labor/obstructed labor, n (%)	39 (5.4%)	20 (5%)	19 (5.9%)	.603
Uterine ruptures, n (%)	16 (2.2%)	0 (0%)	16 (4.9%)	<.001
Abortion complications, n (%)	2 (0.3%)	0 (0%)	2 (0.6%)	.199
Premature live births, n (%)	17 (2.3%)	10 (2.5%)	7 (2.2%)	.768
Other complications				
Hemorrhage, n (%)	47 (6.5%)	24 (6%)	23 (7.1%)	.545
Pre-eclampsia/eclampsia, n (%)	4 (0.6%)	2 (0.5%)	2 (0.6%)	>.999
Puerperal infections, n (%)	56 (7.7%)	16 (4%)	40 (12.3%)	<.001
Obstetric complications treated or referred, n (%)	87 (12%)	33 (8.2%)	54 (16.7%)	.001
C-sections performed, n (%)	245 (33.8%)	151 (37.7%)	94 (29%)	.014
Death following abortion, n (%)	0 (0%)	0 (0%)	0 (0%)	-
Live births < 2500 g, n (%)	22 (3.0%)	13 (3.2%)	9 (2.8%)	.717

⁎ Composite comprised of maternal deaths, stillbirths registered, and neonatal deaths (0 to 28 day). Prepilot, January 2021 to February 2022; During pilot, March 2022 to February 2023.

## Structured discussion

### Principal findings

We found that the mMIST intervention was feasible and highly acceptable to maternal health providers who staffed or used the system. Feasibility was primarily assessed via demand and satisfaction. The demand increased over time, averaging 14 calls per month. This demand level met our established benchmark of 1 call every 2 days. The acceptability level of 95% was also higher than our pre-established 70% level among providers. We also demonstrated that it was feasible to obtain and evaluate administrative data on relevant neonatal and maternal outcomes. However, we observed mixed results regarding the frequency of outcomes when comparing the pilot period with the year preceding the pilot, most likely due to the sub-optimal quality of the administrative data collection.

### Results in context

Our findings on feasibility of implementing the mMIST and acceptability of the intervention support a growing body of evidence suggesting that in LMICs, provider to provider mHealth support systems are useful and increasingly becoming a part of the care landscape.^8^^,^^15^^,^^16^ Our findings also align with successful efforts to build large scale sustainable provider to provider advice systems such as in the Safe Births Bundle of Care program in Tanzania, which began similar formative and pilot process.^24^ In addition, the promise and feasibility of implementing the mMIST in the Ndop health District are similar to promising mHealth findings for a variety of health outcomes in other regions of Africa including Botswana,^25^ Ethiopia,^10^^,^^16^ Nigeria,^6^ and Rwanda.^8^ However, most of these mHealth studies targeted pregnant women to improve education, data collection, prenatal and antenatal care attendance, and medication adherence; and only a few also addressed capacity building and decision support aids for maternal health providers.^6^^,^^10^

Phone connectivity network issues and electricity disruption identified as structural barriers to optimal implementation of the mMIST in our pilot study are also similar to structural barriers identified in previous studies.^7^^,^^18^^,^^26^ Feasibility of our pilot study was enhanced by our deliberate efforts to implement lessons learned from previous studies regarding barriers to mHealth interventions in LMIC. Lessons on the need to partner with local nongovernmental, governmental and cell phone companies^14^ were incorporated into the formative work for the mMIST and in the implementation of the pilot study. The mMIST intervention leveraged the ongoing ALSO national training program run by our country partner CBCHS in which maternity workers are trained and certified to handle obstetric emergencies. By using this approach, we contributed to capacity building among maternity health workers and the likelihood of sustainability if successful.

### Clinical implications

The primary focus was on the feasibility of implementing the mMIST intervention to prepare for a larger scale testing of the intervention. Additional research is required before findings can be confidently used to improve clinical outcomes. Furthermore, our mMIST intervention addressed identified structural barriers through capacity building to bolster the knowledge and skills of first-line responders, staff, and providers. We also relocated the mMIST exchange to a taller building and added back up solar power to ensure optimal function. By linking a network of peripheral, first line and expert providers sharing life-saving evidence-based care, the efficiency and quality of care was potentially enhanced.^18^ We also identified important gaps in the accuracy of data collection that will need to be addressed through data collection optimization to more validly evaluate the impact of mMIST.

### Research implications

This pilot work provided the foundation for a roll out and evaluation of mMIST in the Northwest region of Cameroon.

### Limitations and strengths

Findings of this pilot study must be interpreted with caution. The study was conducted only in one health district and these findings may not represent other districts in the Northwest region or in the country. Also, half of the calls came from one referral facility, further limiting the generalization of findings. In addition, the primary focus was on feasibility to prepare for a larger scale testing of the intervention. Further evaluation in multiple districts is now in progress. The use of broad categories in the reasons for mMIST calls, such as the lack of specific details on types of infections, also limits the usefulness of findings. In addition, since the administrative data at the district level were provided in aggregate, cases where both maternal and neonatal deaths occurred were counted as separate events in the composite outcome. The use of administrative data with marked variability over time and patterns suggesting biased reporting likely related to COVID and ongoing civil strife, limited the inferences that could be made on changes in patient outcomes. Despite these limitations, our findings on the feasibility of implementing the mMIST and obtaining administrative outcomes data are important and paved the way to better designed studies evaluating the real-world effectiveness of the mMIST system.

## Conclusions

This study was part of a process of adaptation and evaluation of mMIST. Adaptation to local context, implementation approach, and engagement of, and buy-in from multiple stakeholders in our formative work contributed to feasibility and promising findings in this pilot. Paying attention to contextual influences can allow for the full potential of mHealth intervention to be harnessed to improve perinatal health outcomes. mMIST is a promising solution for healthcare improvement in LICs that is geographically scalable and if successfully evaluated could potentially be applicable to disciplines other than pregnancy to improve health outcome.

## CRediT authorship contribution statement

**Comfort Enah:** Writing – review & editing, Writing – original draft, Validation, Supervision, Resources, Project administration, Methodology, Investigation, Funding acquisition, Formal analysis, Conceptualization. **Victoria Jauk:** Writing – review & editing, Writing – original draft, Validation, Project administration, Methodology, Formal analysis, Data curation, Conceptualization. **Mary Glory Ngong:** Writing – review & editing, Validation, Supervision, Project administration, Investigation, Data curation, Conceptualization. **Christyenne Lily Bond:** Writing – review & editing, Validation, Supervision, Project administration, Methodology, Formal analysis, Data curation, Conceptualization. **Lionel Neba Ambe:** Writing – review & editing, Validation, Supervision, Resources, Methodology, Data curation, Conceptualization. **Rahel Mbah Kyeng:** Writing – review & editing, Supervision, Project administration, Investigation, Data curation, Conceptualization. **Roland Mbole:** Writing – review & editing, Software, Resources, Methodology, Data curation, Conceptualization. **Waldemar A. Carlo:** Writing – review & editing, Validation, Methodology, Investigation, Funding acquisition, Conceptualization. **Gregory Halle-Ekane:** Writing – review & editing, Validation, Supervision, Methodology, Investigation, Conceptualization. **Pius Muffih Tih:** Writing – review & editing, Supervision, Methodology, Investigation, Funding acquisition, Data curation, Conceptualization. **Jeff Szychowski:** Writing – review & editing, Supervision, Methodology, Investigation, Funding acquisition, Conceptualization. **Henna Budhwani:** Writing – review & editing, Writing – original draft, Supervision, Methodology, Investigation, Funding acquisition, Conceptualization. **Alan Thevenet N. Tita:** Writing – review & editing, Writing – original draft, Validation, Supervision, Methodology, Investigation, Funding acquisition, Formal analysis, Conceptualization.

## Figures and Tables

**Table:** 

	Disagree	Neither disagree nor agree	Agree
I understand the purpose of mMIST.	ο	ο	ο
mMIST will be easy to use.	ο	ο	ο
mMIST will be helpful in providing care to patients.	ο	ο	ο
I am confident in my ability to use mMIST.	ο	ο	ο
I have all the equipment I need to use mMIST.	ο	ο	ο
The training was good.	ο	ο	ο
I am satisfied by the training.	ο	ο	ο
The training answered my questions about mMIST.	ο	ο	ο

## Appendix A mMIST satisfaction survey

Q1 Please rate the following statements:

Q2 Please list ways we could improve mMIST.

——————————————

——————————————

Q3 Is there anything else you would like to share?

——————————————

——————————————
